# Target Salt 2025: A Global Overview of National Programs to Encourage the Food Industry to Reduce Salt in Foods

**DOI:** 10.3390/nu6083274

**Published:** 2014-08-21

**Authors:** Jacqui Webster, Kathy Trieu, Elizabeth Dunford, Corinna Hawkes

**Affiliations:** 1 The George Institute for Global Health, Food Policy Division, World Health Organization Collaborating Centre on Population Salt Reduction, Sydney 2050, Australia; E-Mails: ktrieu@georgeinstitute.org.au (K.T.); edunford@georgeinstitute.org.au (E.D.); 2 World Cancer Research Fund, Policy and Public Affairs, London WC1B 3HH, UK; E-Mail: c.hawkes@wcrf.org

**Keywords:** salt reduction, sodium, food composition, government initiative, international, cardiovascular diseases, food industry, monitoring

## Abstract

Reducing population salt intake has been identified as a priority intervention to reduce non-communicable diseases. Member States of the World Health Organization have agreed to a global target of a 30% reduction in salt intake by 2025. In countries where most salt consumed is from processed foods, programs to engage the food industry to reduce salt in products are being developed. This paper provides a comprehensive overview of national initiatives to encourage the food industry to reduce salt. A systematic review of the literature was supplemented by key informant questionnaires to inform categorization of the initiatives. Fifty nine food industry salt reduction programs were identified. Thirty eight countries had targets for salt levels in foods and nine countries had introduced legislation for some products. South Africa and Argentina have both introduced legislation limiting salt levels across a broad range of foods. Seventeen countries reported reductions in salt levels in foods—the majority in bread. While these trends represent progress, many countries have yet to initiate work in this area, others are at early stages of implementation and further monitoring is required to assess progress towards achieving the global target.

## 1. Introduction

At the World Health Assembly in May 2013, all Member States of the World Health Organization signed up to the target to reduce salt intake by 30% by 2025 [1]. Salt reduction has been shown to be one of the most cost-effective, and in some cases, cost-saving ways of reducing the growing burden of non-communicable diseases, primarily through reducing the incidence of cardiovascular diseases and strokes [2]. The physiological requirement for salt is less than 1 gram per day [3], however, most populations are eating between 9 and 12 grams [4]. Excess salt intake progressively elevates blood pressure levels throughout life, which greatly increases the risks of vascular disease [5,6] and is likely responsible for about half of the disease burden ascribed to high blood pressure [7]. In the US, it is projected that an intervention reducing salt by 3 g/day would save between 10 and 24 billion dollars in health costs each year [8]. Similarly high levels of health care savings have been found in other countries—including low and middle income economies.

The contribution of different foods to salt in the diet varies across the globe, with as much as 95% of dietary salt estimated to come from processed foods in the UK compared to 63% in Japan (20% from soy sauce alone). In contrast, as much as 76% of dietary salt comes from home cooking in the South of China [9]. Whilst the relative proportion of each differs from country to country processed products contributing to salt in the diet usually include bread, breakfast cereals, processed meats, dairy products, soups and sauces (particularly soy sauce and fish sauce in many Asian countries), biscuits and salty snacks [10]. Where processed foods contribute significantly to salt in the diet it means that, even if people try to reduce their salt intake by not adding salt, they will still consume large amounts in the foods that constitute their usual diets. This means it is very difficult to reduce population salt intake by approaches that seek to change consumer behavior because the salt that is obvious to individuals (the salt that they add during cooking or at the table) is only a fraction of their daily consumption [11]. The most effective means of reducing salt consumption in these cases is to reduce the salt content of manufactured and catered foods as this does not rely on changes to consumer behavior [4,12]. The WHO and numerous national and regional government advisory groups, including the United Kingdom’s Scientific Advisory Group on Nutrition, The United States Institute of Medicine and the Pan American Health Organization, have therefore been encouraging manufacturers to reduce the salt levels in processed foods for many years [13,14,15] and specific examples of countries who have taken this route are well reported, including Finland and Japan in the 1970s and the UK in the 2000s [16,17].

The objective of this paper is to provide a systematic overview of national approaches to working with the food industry to reduce salt in foods with a view to identifying which types of program are more likely to have an impact. It is the first comprehensive review of this kind and provides insights into future approaches to working with the food industry as part of national strategies to reduce population salt intake.

## 2. Methods

### 2.1. Search Strategy

As part of a broader review of progress on national salt reduction initiatives, this study identified and documented programs to engage the food industry to reduce salt in foods by:

- Systematically reviewing peer-reviewed and grey literature and consulting international experts.
- Recording programs in a database based on pre-defined criteria.
- Sending questionnaires to country program leaders to supplement/verify information.

The systematic search of the peer-reviewed if the literature included the following databases: Cochrane Central Register of Controlled Trials (CENTRAL), Cochrane Public Health Group Specialized Register, MEDLINE, EMBASE, Effective Public Health Practice Project Database, Web of Science, TRoPHI databases and LILACS database. Search terms used included “salt”, “sodium”, “strategy”, “intervention”, “initiative” or “government”. A targeted search for pertinent grey literature documents was conducted in OpenGrey, Google, World Health Organization and regional office databases and websites, governmental websites (e.g., Food Standards Agency, Public Health Agency of Canada, Centers for Disease Control and Prevention), scientific or non-governmental organization websites (e.g., Institute of Medicine, Food Safety Authority of Ireland, Heart Foundation) and international or national salt reduction associations (e.g., World Action on Salt and Health).

A database of initiatives on salt reduction initiatives was established based on the information identified. Details were verified through questionnaires sent to country contact people. Information on programs to engage the food industry to reduce salt in foods was then extracted and analysed.

### 2.2. Inclusion and Exclusion Criteria

National strategies were defined as having government involvement and either a written strategy or one or more specific initiatives focused on reducing salt. Programs of work with the food industry were defined as either a series of meetings or established forum, or the development of voluntary or regulatory targets for salt levels in foods for the industry to achieve in a given timescale. Government statements or policies stating that the food industry should reduce salt in foods were not viewed as engagement with the food industry to reduce salt in foods.

### 2.3. Data Extraction and Analysis

For each national program of work with the food industry identified, key characteristics were documented. This included whether the program was led by government, NGO or industry, whether it involved industry meetings or the development of targets, whether they were voluntary or legislated, how the program was monitored, and if there had been any impact. The programs were then analysed in relation to each of these characteristics. In countries where there were multiple sources of data documenting change in sodium levels of foods, data from the most accurate method of monitoring sodium levels was reported.

## 3. Results

### 3.1. Approach to Working with the Industry and Product Categories

Out of the 83 countries that were identified with salt reduction strategies in place or planned, 59 countries reported programs of work with the industry to reduce salt in foods. Twelve others reported future plans in this area. Approaches to working with the food industry vary between negotiating commitments through industry meetings, agreeing voluntary targets for specific product categories, or establishing mandatory limits (legislation).

Twenty three out of the 59 countries reported industry meetings and 38 had established voluntary and/or mandatory sodium content targets. Thirty five countries established voluntary targets. Out of these, twenty two had voluntary targets for multiple food products (15 in Europe, 3 in the Americas and 4 in the Western Pacific Region), eight had voluntary targets just for bread, four countries had targets for bread and processed meats and one country had targets just for dehydrated soups and sauces. Nine countries had established mandatory sodium targets, all of which had a target for bread. Two countries (South Africa and Argentina) had mandatory targets for a range of food products, two countries had mandatory targets for up four product categories and five countries (all in Europe or the Americas) had mandatory targets for bread only (See Table 1).

**Table 1:** National approaches to working with the food industry to reduce salt in foods.

Region	Voluntary Targets (Multiple Products)	Voluntary Targets (Bread Only)	Mandatory Targets	Industry Meetings	Reformulation Planned
Africa			South Africa		Mauritius
Americas	Brazil Canada Ecuador USA	Chile Mexico Uruguay	Argentina Paraguay (bread)	Barbados Costa Rica Cuba	Colombia Costa Rica (Voluntary Targets)
Eastern Mediterranean				Morocco	Iran
Europe	Austria Belgium Bulgaria Czech Republic Denmark Finland Greece Hungary Ireland Israel Netherlands Poland Portugal Slovenia Spain Sweden Turkey UK	Croatia France Italy Lithuania	Belgium (bread) Bulgaria (bread, cheese, meat products, lutenica) Greece (bread, tomato products) Hungary (bread) Netherlands (bread) Portugal (bread)	Cyprus France Italy Latvia Luxembourg Montenegro Norway Romania Slovakia Switzerland (bread)	Estonia Malta
Eastern Mediterranean				Morocco	Iran
South East Asia				Bangladesh * Indonesia Sri Lanka Thailand	Sri Lanka (Mandatory targets)
Western Pacific	Australia Cook Islands Fiji Korea New Zealand *	Mongolia		Federated States of Micronesia Japan Malaysia New Caledonia Singapore	French Polynesia Guam Samoa Solomon Islands Tonga Vanuatu Viet Nam

* Non-governmental organization leading reformulation.

### 3.2. Mechanisms for Monitoring

There are a number of different approaches to monitoring salt levels with varying degrees of comprehensibility, objectivity, accuracy and resource intensiveness. At the least objective and resource intensive end of the spectrum, food companies can be asked to report on the changes they have made (industry self-report). In the middle, regular food label surveys can be undertaken to establish baseline salt levels [18] and monitor changes [19,20] and branded food composition databases can be established to ensure progress by food companies can be evaluated [20]. At the other end of the spectrum chemical analysis of food composition is the most accurate and transparent way of monitoring changes to sodium levels in foods.

Forty four of the 59 countries with food industry programs reported having established monitoring mechanisms. The most frequently reported approach was chemical analysis of food (15), or chemical analysis with some other form of monitoring: chemical analysis of foods and databases (8), chemical analysis and industry report (5) or chemical analysis of foods and survey of food labels (2). Ten countries reported using just other forms of monitoring: database (3), food label surveys (2) and industry self-report (5). Three countries indicated that they were using three methods to monitor salt levels in foods. One country (Ecuador) indicated it was monitoring progress but did not specify a mechanism.

### 3.3. Documented Changes in Salt Levels

Seventeen countries (nine in Europe, four in the Americas and four in the Western Pacific Region) reported a reduction in salt levels in one or more product categories and all except Malaysia include a reduction in bread. Five countries (France, Ireland, Malaysia, the UK and USA) reported reductions across a wide range of product categories. Canada, Finland, Italy and Australia reported reductions in three product categories. The Netherlands reported reductions in salt levels in bread and canned vegetables. The other seven countries reported reductions just in bread (See Table 2).

All of the reductions that were reported were where there were targets for salt levels for that particular food category. The majority were voluntary targets. Argentina, Belgium and the Netherlands reported reductions where mandatory targets were in place.

The majority of the reductions reported (68%) were based on chemical analysis of food, except for Austria, Italy and Malaysia which were based on industry self-report, and Australia, Canada and the UK where reductions were based on product surveys (See Table 2).

**Table 2:** Impact of programs of industry engagement on salt levels in foods.

Country	Approach	Method	Reduction in Salt Levels	Timeframe
Argentina	V *	Food analysis	Bread: 18% [21]	2009–2010
Australia [22]	V	Product label survey	Bread: 9% Breakfast cereals: 25% Processed meats: 8%	2010–2013
Austria	V	Industry self-report	Not across the sector-surveyed 112 bakeries and found 30 tonnes of salt reduced in bread/pastry	2011–2013
Belgium	M	Food analysis	Bread: 6% [23]	1990–2009
Canada	V	Product label survey	Not across the sector-small survey of sodium levels on labels found a 11% reduction in pantry breads, 14% in breakfast cereals and 8% in canned soups	2009–2011
Chile	V	Food analysis	Bread (maraquetta): 38% [24]	2010–2012
Finland	V	Food analysis	Bread: 20% [25] Meat products, cheese and ready meals: 20%–25% [26]	1990s–2009
France [27]	V	Food analysis	Bread: 12% Pizzas & quiches: 23% Soups: 32% Mixed dishes: 17%	2008–2011 2003–2011 2003–2011 2003–2011
Ireland [28]	V	Food analysis	White Bread: 18% Wholemeal bread: 29% Breakfast cereals: 30% in biscuit based cereals to 59% in cornflake based cereals Fresh and packet soups: 12% and 19% respectively Cooking sauces: 35% in Bolognese sauce to 71% in black bean sauce Butter: 18%	2003–2013 2003–2011 2005–2013 2004–2012 2007–2011
Italy	V (bread)	Industry self-report (currently being analyzed)	Reductions in baking products, sauces, processed meats (salami, ham)	2013
Malaysia	IM	Industry self-report	Not across the sector. 30 high in salt food items have reduced salt content by 1% to 40% Biscuit categories: 11%–35% Tomato sauce: 9.5%–40% Instant noodles: 2%–20% Flavorings cubes/powder: 1.1%–16% Frozen meat: 13%	2011–2012
Mongolia	V (bread)	Food analysis	Bread: 12%	2011
Netherlands	M (bread)	Food analysis	Bread: 9% Canned vegetables also reduced	2009–2012
New Zealand	V	Food analysis	Bread: 22% [29]	1987/88–2009
Spain	V	Food analysis	Bread: 26% [30]	2005–2009
United Kingdom	V	Product label survey and industry self-report	Bread: 20% [31] Federation of Baker sliced breads (80% of bread sold in UK): >30% [32] Branded breakfast cereals: 49% [32] Pasta sauces and soups: 29% and 25%, respectively [32] Sweet and savoury biscuits: 45% and 25%, respectively [32] Cakes: 25% [32] Pastries: 40% [32] Crisps, extruded and pelleted snacks: 13%, 32% and 27%, respectively [32]	2001–2011 1980s–2008 1998–2007 2003–2005 2006–2008 2006–2008 N/A 2007
United States of America [33]	V	Database (food analysis)	White Bread: 29% Butter: 28% Frozen peas: 81% Canned tuna: 50% Canned chicken soup: 35% Salad dressing: 21% Pretzels: 19% Ham: 14% Corn flakes: 5%	1963–2007

V: Voluntary sodium targets established; M: mandatory sodium targets established; IM: negotiating commitments to lower sodium through industry meetings. * Argentina’s targets became mandatory in 2012, after the period of change reported here.

### 3.4. Extent of Reductions

Reductions ranged from 6% (Belgium) to 38% (Chile) in breads with the UK, Canada, New Zealand and Ireland all also reporting between 20% and 30% reductions in breads. For other product categories, reductions range from 5% (cornflakes, USA) to 81% (frozen peas, USA) (See Table 2).

## 4. Discussion

This is the first comprehensive global overview of progress to work with the food industry to get companies to reduce salt in foods. The number of countries reporting food industry has more than doubled since a previous review in 2010 [17]. Fifty nine countries, 80% of the countries with national strategies to reduce salt, now include programs of work with the food industry.

The approach to industry engagement is one of the key differentiating factors between salt reduction initiatives, with some countries establishing mandatory targets, whilst the majority chose voluntary agreements and others negotiated commitments through meetings. Over 60% of the food industry programs identified (38 countries) have established product-specific targets for salt levels in foods. Whilst most of these are voluntary targets, nine countries have established legislation—seven just for bread, or for bread and other food categories. Two countries, South Africa and Argentina, have recently established legislation for a wide range of product categories.

All of the countries that have demonstrated an impact have set targets for salt levels in foods except for Malaysia, which just negotiated the reductions through industry meetings. Mostly the targets were voluntary, although three countries (Netherlands, Belgium and Argentina) reported reductions in salt levels in breads as a result of mandatory targets. Other countries with voluntary and legislated targets had yet to report on progress.

Many public health experts believe regulation is a much stronger driver for industry reformulation than voluntary agreements as it usually carries a penalty for non-compliance [34,35]. Yet many voluntary programs seem to be making progress. That said, whilst the targets for salt levels in foods in Finland are currently voluntary, the effectiveness of Finland’s salt reduction program is often attributed to the introduction of legislative requirements to label the salt content of foods in the 1980s. Products containing more than a specified amount of salt had to carry a high salt warning, which resulted in food companies reformulating products to avoid this and some high salt products disappearing off the market altogether [36]. Likewise, the success of the UK initiative, whilst based on voluntary agreements, is attributed to sustained strong government leadership and pressure, reinforced by robust monitoring mechanisms, and underpinned by active NGOs [17,32].

In some countries laws are difficult to introduce, taking a considerable amount of time to move through bureaucratic processes. They may also be less flexible, so that once a law is established it’s difficult to change. Had the UK Food Standards Agency targets been enshrined in law initially, for example, it would have taken longer to revise them once it became apparent that the food industry was going to be able to make larger reductions in a shorter timescale. For this reason, many countries prefer to adopt voluntary approaches, establishing partnerships with the food industry to negotiate agreements and ensuring adequate monitoring mechanisms are in place to hold the food industry to account. Often the penalty for non-compliance in these cases is the threat of negative publicity when surveys highlighting the salt content of foods are published [18,19].

On the other hand, arguments in favour of legislation include the fact that fiscal penalties for non-compliance can be introduced, legislation is more difficult to disband if a new government comes into power and it is better at creating a level playing field for the industry as all companies are forced to comply [37]. Most laws for salt levels in foods are relatively newly established so there has been insufficient time to demonstrate an impact. Ongoing monitoring of the legislation in Argentina, South Africa and other countries is now required to demonstrate the feasibility and likely impact of legislation to other countries with a view to supporting uptake in other countries.

Regardless of whether or not the approach taken is voluntary or legislative, one of the key decisions for countries to make is which foods to set salt level targets for. The contribution of processed foods to salt intake varies considerably throughout the world and national strategies to reduce salt need to take this into account. Our review showed that whilst most countries that had established targets had established them for multiple food sources, many (37%) had established targets just for bread or for bread and one other product. In view of the broad range of processed foods that contribute to salt intake in many countries, this is concerning. Whilst bread is a major contributor to salt, contributing as much as 20% or 30% of salt in the diet in some countries, reducing salt in bread alone, even by as much as 30% is not going to achieve the population wide reductions required to achieve the targets of a 30% reduction by 2025. This fact was recognized by the UK Food Standards Agency when it first developed their Salt Model in 2003. Whilst this established that just four food product categories (white bread, bacon and ham, breakfast cereals and homemade meat-based dishes) contributed around 40% of non-discretionary salt to the UK diet, a whole range of other food categories (cheese, sausages, fat spreads, baked beans, milk and cream, other bread, soup, pizza, crisps and savory snacks and meat pies) each contributed between 2% and 4% so the only way to effectively reduce population salt intake was to target this wide range of product categories.

The EU Framework for national salt reduction initiatives also suggests that Member States select at least five categories to focus on and make stepwise reductions seeking to achieve a 16% reduction on average for these food products over four years [38,39].

However, out of the 17 countries that had demonstrated an impact, only five countries reported reductions across a wide range of product categories. Canada, Finland, Italy and Australia reported reductions in three product categories. The Netherlands reported reductions in salt levels in bread and canned vegetables. The other seven countries reported reductions just in bread. Whilst it may have not been possible to capture all the reductions made through the research undertaken here, it seems clear that more countries will need to get the food industry to reduce salt in a greater number of product categories to achieve subtle long-term changes in consumer preferences for less salty foods and to maximize the opportunity for achieving the global targets for salt reduction.

Most (73%) of the countries had mechanisms in place for monitoring the progress of industry salt reduction programs, with analysis of food composition to determine sodium content being the most frequently reported monitoring mechanism (73%). Out of the 17 countries that had demonstrated an impact, 14 were based on analysis of food composition. Whilst food analysis is undoubtedly the most accurate method of assessing individual food products, it can be expensive to do comprehensively. Key elements required for this type of monitoring are that it be objective and transparent in both methods and outcomes, be undertaken in a systematic way on a regular basis and that it be quantitative in nature. For the purpose of improving the nutritional composition of the food supply there are some key issues that need to be addressed—data accuracy and reliability, the ability to update information regularly, the ability to access data easily and affordably, completeness of coverage, independent third-party monitoring and brand/product-specific information.

Whilst not reported on in this research, the Global Food Monitoring Group (FMG) has begun work to collate data on the nutritional composition of processed foods in multiple countries using comprehensive large scale product label surveys [20] with the aim of objectively and transparently monitoring changes in the nutritional composition of processed foods globally. Currently, the FMG has representation from 31 countries, with over 200,000 products (both packaged and processed at the barcode level and food service items) included (68). Data have already been used by public health researchers to show the often wide variation in sodium levels in foods [40,41,42,43].

Australia, Canada and the UK are all members of the FMG and have reported reductions across three, five and a broad range of product categories respectively, based on repeated comprehensive product label surveys. Comprehensive surveys of product labels can be done relatively easily at low cost and, in developed countries, can be a reasonably reliable method of monitoring changes in the nutritional composition of foods on a larger scale than what is possible with chemical analysis. Available research suggests that the NFP labels are accurate in approximately 80% of products, with inaccurate cases generally containing less risk-associated nutrients (such as sodium and saturated fat) than the specified value on the label [44,45,46]. Whilst product sales data could provide more insight into the precise impact of changes in salt content of different food products on population salt intake, these data are usually expensive to access and have limits on usage and sharing of information, and comprehensive branded information across the whole product category is provides and adequately robust and more feasible assessment [47].

Where monitoring is based on food analysis it is not always clear, from the data reported by countries, whether sodium reductions have been made across the board or just for some brands in the category. Whilst it is encouraging if some brands, particularly leading brands, are making changes in the right direction, it is important to make sure that changes are being made across the board as far as possible. The FMG approach provides an alternative approach to doing this and it is important that the public health researchers responsible for these surveys are linked with the government representatives responsible for salt reduction strategies to ensure that larger scale product label surveys can be used in parallel with food composition analysis where appropriate.

In fact, building on that theme of cross-sector collaboration, whilst a key strength of the research here is the systematic comprehensive approach, one limitation is that national surveys of this kind rely on the point of view of one country representative. Whilst in most cases they are responsible for the government strategy, they do not always have a complete overview of all of the relevant work in the country, or the perspectives of different stakeholders in the complex food policy environment. In future, multi-stakeholder research would be useful to supplement some of the survey findings. Such research could also help to establish a deeper understanding of what the main factors that contribute to the effectiveness and sustainability of different programs are. This could include technical limits on salt reduction and the potential use of salt substitutes to overcome this, the nature of marketing of lower salt content, the role of NGO and advocacy organisations in both lobbying and monitoring progress, and the potential for international government and food industry agreements to support progress.

This review has focused on engagement with the food industry to reduce salt in foods. However, most salt reduction strategies are multi-faceted and comprise of parallel programs to change consumer attitudes and behaviours relating to salt often backed by clearer front of pack labeling. The relative impact of these different elements and how they inter-relate with each other will be the subject of a subsequent review by the authors. Integrating salt reduction into broader nutrition education, health promotion and non-communicable disease prevention programs [48] should also be a priority, particularly where resources are scarce and there is limited workforce capacity.

## 5. Conclusions

Countries are increasingly recognizing the health benefits of salt reduction and establishing programs to encourage the food industry to take salt out of foods. Many countries are already reporting reductions in salt levels as a result of the establishment of targets for salt levels in foods. Whilst most programs are still voluntary, quite a few countries have so far introduced legislation for bread, or bread and one other product, and South Africa and Argentina have recently introduced legislation for a wide range of products. Robust, comprehensive monitoring of the nutritional composition of processed foods is vital to ensure that salt reduction pledges by industry are being upheld, particularly if done under a voluntary framework. Whilst almost one third of the countries with programs in place have already demonstrated an impact, most programs are at early stages of implementation, the scope of existing programs will need to be widened and many countries still need to develop programs to ensure the global target of a 30% reduction by 2025 can be achieved, this in turn will prevent millions of avoidable deaths globally and reduce the health care budgets of countries by many millions of dollars each year.
